# Macrophage autophagy in macrophage polarization, chronic inflammation and organ fibrosis

**DOI:** 10.3389/fimmu.2022.946832

**Published:** 2022-10-06

**Authors:** Jun-Hao Wen, Dong-Yi Li, Shan Liang, Chen Yang, Ji-Xin Tang, Hua-Feng Liu

**Affiliations:** Guangdong Provincial Key Laboratory of Autophagy and Major Chronic Non-Communicable Diseases, Key Laboratory of Prevention and Management of Chronic Kidney Disease of Zhanjiang, Institute of Nephrology, Affiliated Hospital of Guangdong Medical University, Zhanjiang, China

**Keywords:** macrophage, autophagy, macrophage polarization, fibrosis, chronic inflammation, LC3-associated phagocytosis

## Abstract

As the essential regulators of organ fibrosis, macrophages undergo marked phenotypic and functional changes after organ injury. These changes in macrophage phenotype and function can result in maladaptive repair, causing chronic inflammation and the development of pathological fibrosis. Autophagy, a highly conserved lysosomal degradation pathway, is one of the major players to maintain the homeostasis of macrophages through clearing protein aggregates, damaged organelles, and invading pathogens. Emerging evidence has shown that macrophage autophagy plays an essential role in macrophage polarization, chronic inflammation, and organ fibrosis. Because of the high heterogeneity of macrophages in different organs, different macrophage types may play different roles in organ fibrosis. Here, we review the current understanding of the function of macrophage autophagy in macrophage polarization, chronic inflammation, and organ fibrosis in different organs, highlight the potential role of macrophage autophagy in the treatment of fibrosis. Finally, the important unresolved issues in this field are briefly discussed. A better understanding of the mechanisms that macrophage autophagy in macrophage polarization, chronic inflammation, and organ fibrosis may contribute to developing novel therapies for chronic inflammatory diseases and organ fibrosis.

## Introduction

As a leading cause of morbidity and mortality, fibrosis is the common pathway of various chronic inflammatory diseases in organs and causes a nearly 50% death rate in patients in developed countries (1, 2). Inflammatory monocytes and tissue-resident macrophages are the important regulators of organ fibrosis (3). The injury of tissues can induce an inflammatory response, causing the recruitment, proliferation, and activation of a variety of immune cells, such as neutrophils and macrophages, to contribute to tissue repair (4, 5). When the injury is mild, the inflammatory response will resolve quickly, and the function of the organ can be fully restored. However, if the injury is severe or there are repeated injuries, the chronic inflammation will persist, which can result in organ fibrosis, gradually losing the normal function of tissue and ultimately causing organ failure and even death of the organism (6). Therefore, inflammatory responses in tissues need to be tightly regulated so as to restore tissue function and prevent chronic inflammation and fibrosis. Among the various immune cells involved in organ fibrosis, macrophages have been shown to be a major player in chronic inflammation and fibrosis (3). Because of the important roles of macrophages in chronic inflammation and fibrosis, there has been a great deal of interest in the past few years in studying the role of different types of macrophages in organ fibrosis.

As an important self-degrading system in eukaryotic organisms, autophagy plays an essential role in sustaining normal energy supply during critical periods of development and in response to nutritional stress (7). Besides, autophagy also plays an essential in maintaining cellular homeostasis by eliminating misfolded or aggregated proteins, clearing damaged organelles such as mitochondria (8), endoplasmic reticulum (9) and lysosomes (10), and removing pathogens within cells (11). In addition, autophagy is also involved in cell senescence (12), antigen presentation (13), genomic instability (14), apoptosis (15), and ferroptosis (16). Therefore, the dysregulation of autophagy is associated with many human diseases, such as inflammation, aging, metabolic diseases, neurodegenerative disorders, and cancers (17–19).

Macrophages, a class of highly heterogeneous immune cells, can polarize to various phenotypes stimulated by the surrounding microenvironment (20). It is now known that macrophage polarization determines the fate of an organ during inflammation or injury. When an organ or a tissue suffers from an infection or injury, macrophages are first polarized to the proinflammatory M1 phenotype to release proinflammatory cytokine to aid the removal of antigens and necrotic cells. At the repair stage, the M1 macrophages need to polarize with the M2 macrophages, which can secrete anti-inflammatory cytokines to suppress the inflammation, and promote tissue repair and remodeling. However, if the pro-inflammatory macrophage persists, this can result in the continuous production of proinflammatory factors, causing chronic inflammation and ultimately the progression of organ fibrosis.

Autophagy can regulate the polarization of macrophages (21–26). Macrophage autophagy alleviates chronic inflammation and the progression of organ fibrosis by inhibiting M1 pro-inflammatory macrophage polarization. However, the specific molecular mechanism by which autophagy affects macrophage polarization remains unknown. In this review, we will discuss the current understanding of the function of macrophage autophagy in macrophage polarization, chronic inflammation, and various organ fibrosis, highlight the function of macrophage autophagy in chronic inflammation and fibrosis in different organs, such as lung and kidney, and finally briefly discuss the remaining questions in this area. A better understanding of the mechanisms that macrophage autophagy in macrophage polarization, chronic inflammation, and organ fibrosis may contribute to developing novel therapies for chronic inflammatory diseases and organ fibrosis.

## Autophagy in macrophage polarization

### Classification and function of autophagy

**Figure 1:**
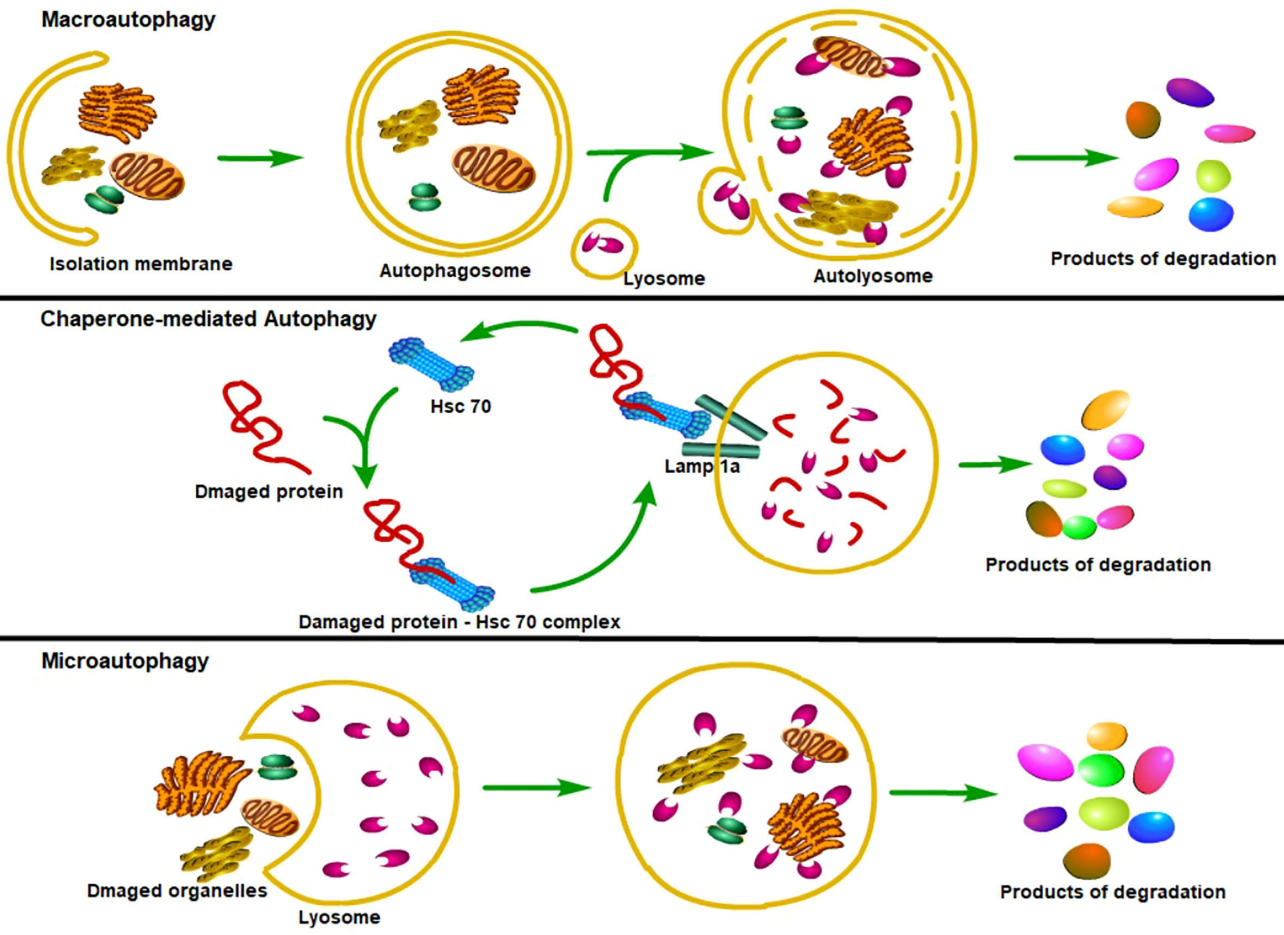
Classification of autophagy. Major types of autophagy. According to the way that eukaryotic cells deliver cytoplasmic cargo to lysosomes for degradation, autophagy can be divided into three major types. First, macroautophagy could both selectively and non-selectively engulf bulk cytoplasmic components by sequestering these cargoes to a specialized double-membrane vesicle known as the autophagosome; autophagosome is then fused with the lysosome, where the cargo is degraded and the resulting macromolecules are released into the cytosol for reuse. Second, the CMA only degrades soluble proteins in a selective manner through the Lamp1a receptor on the lysosome to recognize and translocate unfolding proteins. Third, microautophagy refers to the lysosome itself engulfing cytoplasmic material or large structures by invading the lysosome membrane.

Until now, three major types of autophagy have been reported (27). The first type of autophagy is macroautophagy (hereafter referred to as autophagy), which can sequester the cellular materials into a double-membraned vesicle—autophagosome, autophagosome then fuses with the intracellular lysosomes to form autophagolysosomes, where substances in the autophagosome are degraded and reused (28). Due to the difference in inducing factors, the autophagosomal cargo can be sequestered in a nonselective manner (bulk autophagy) or in a tightly regulated manner (selective autophagy) (29–32). Another major lysosomal degradation process is chaperone-mediated autophagy (CMA), which can selectively degrade the cytoplasmic proteins containing KFERQ-like motif with the help of the heat-shock cognate protein HSPA8/HSC70 to maintain cellular proteostasis (33, 34). The third lysosomal degradative process is microautophagy, which can directly engulf cytoplasmic cargo, such as the KFERQ-flagged proteins or cytoplasmic contents, through endosomal or lysosomal membranous invaginations (35–39), in an ESCRT (Endosomal Sorting Complexes Required for Transport) proteins-dependent or ESCRT proteins-independent manner (37, 40, 41) (**Figure 1**).

Degradation of damaged organelles and long-lived proteins to maintain cellular homeostasis is the basic function of autophagy, therefore, almost all eukaryotic cells have some degree of autophagy (29, 42, 43). However, the function of autophagy is not just to eliminate the cellular materials, it also functions as a dynamic recycling system producing new building blocks and energy for cellular repair and homeostasis (43). When eukaryotic cells are subjected to intracellular and extracellular stimuli, such as starvation and injury, intracellular autophagy level is significantly increased in response to these stimuli to maintain intracellular homeostasis. Mice with systemic autophagy deficiency experienced perinatal death due to the inability to tolerate post-natal starvation (44–46), suggesting that the presence of autophagy promotes cells or organisms to have the ability to maintain viability under stressed conditions, such as nutrient deficiency. Besides, cell-specific or tissue-specific autophagy deficiency mouse models have shown that autophagy is involved in many diseases, including fibrosis (47–51). Furthermore, autophagy also plays an essential role in aging and longevity; lifestyle changes, such as calorie restriction and physical exercise, have been proven to promote the life span of organisms by stimulating autophagy in organisms (52–54).

### Macrophage autophagy and macrophage polarization

As highly heterogeneous and plastic cells, macrophages play an essential role not only in physiological conditions but also in chronic inflammation and fibrosis (20, 55–57). The activated macrophages have often been simply divided into two groups, the classically activated (or pro-inflammatory) macrophage M1 and the alternatively activated (or anti-inflammatory) macrophage M2 (58). It is now clear that macrophage polarization is a multifactorial process that needs the participation of a number of factors so as to produce different activation scenarios (59). The macrophage phenotype is not fixed, and even if a macrophage adopts a phenotype, it still retains the ability to continue to change in response to new environmental influences. The regulation of macrophage polarization may be a potential therapeutic target in chronic inflammation and fibrosis (60–62).

Autophagy plays an essential role in macrophage polarization (63). Impaired macrophage autophagy can promote macrophage to proinflammatory M1 polarization, which can increase the immune response and lead to hepatic chronic inflammation and injury in obese mice (21). Increasing macrophage autophagy flux *via* ubiquitin-specific protease 19 (USP19) can promote anti-inflammatory M2-like macrophage polarization (24). Small molecule drugs that promote autophagy, can facilitate macrophageto anti-inflammatory M2-like polarization (22, 64–66). For example, docosahexaenoic acid (DHA) promotes M2 macrophage polarization by activating autophagy (66). Spermine, an inducer of autophagy, can inhibit M1 polarization and promote M2 polarization of liver-resident macrophages (Kupffer cells, KCs) in TAA-treated liver (64). In addition, exosomes secreted by cancer cells can also promote the M2-type polarization of macrophages by activating autophagy (25). In conclusion, macrophage autophagy can inhibit macrophage M1-type polarization and therefore alleviating chronic inflammation and organ fibrosis.

## Macrophage autophagy in organ fibrosis

The organs of the body are composed of parenchyma and interstitium. Parenchyma refers to the main structural and functional cells of an organ, such as the hepatocytes in the liver. The interstitium is composed of interstitial cells and the extracellular matrix, such as collagen and proteoglycans. Organ fibrosis refers to the increase of fibrous connective tissue and the decrease of parenchymal cells in organs after sustained or severe injury. Continuous progress may lead to the destruction of organ structure and function, and even failure, which seriously threatens human health and life. Pathologically, organ fibrosis is characterized by the excessive accumulation of extracellular matrix (ECM) such as collagen and fibronectin in an organ due to the imbalance of ECM homeostasis, with increasing deposition and decreasing degradation. Therefore, fibrosis is not exactly a disease, but a result of abnormal tissue repair (67, 68).

Tissue injury can cause tissue cell damage and lead to degeneration, necrosis, and inflammatory response of tissue cells. If the damage is small, the normal parenchymal cells around the damaged cells will undergo proliferation and repair, and this repair can completely restore the normal structure and function. However, when the damage is large or repeated damage and exceeds the regenerative capacity of parenchymal cells around the injury, the connective tissue of interstitial fibers (extracellular matrix) will prolifically repair the defect tissue, that is, the pathological changes of fibrosis will occur. Therefore, fibrosis is essentially a repair response after tissue damage to protect the relative integrity of tissues and organs. The proliferation of fibrous connective tissue repairs the defect, but do not have the structure and function of the original organ parenchymal cells. If this repair response is excessive, strong and out of control, it will cause organ fibrosis and lead to organ function decline. During this process, inflammation plays an essential role and may be a cause of fibrosis (69). Considering macrophage autophagy can inhibit the polarization of macrophages to pro-inflammatory M1 type, it may be a potential target for organ fibrosis (**Table 1**).

**Table 1:** The main experimental evidence and findings about macrophge autophagy in organ fibrosis obtained *in vitro* and *in vivo*.

Treatment	Models	Macrophage autophagy	Type of macrophage	Outcome	*In vitro* or *in vivo*	References
CS	–	AM autophagy↑	AMs	CS-induced PF↓	*In vivo*	(79)
Trehalose	–	AM autophagy↑	AMs	CS-induced PF↓	*In vivo* and *in vitro*	(80, 81)
Dioscin	–	AM autophagy↑	AMs	CS-induced PF↓	*In vivo*	(79, 82)
Dioscin	(Atg5^flox/flox^Dppa3-Cre/+) mice	AM autophagy↓	AMs	CS-induced PF↑	*In vivo*	(79)
MicroRNA-205-5p	–	AM autophagy↑	AMs	CS-induced PF↓	*In vivo* and *in vitro*	(83)
CS	(Atg5^fl/fl^LysM-Cre+) mice	Monocyte-derived macrophage autophagy↓	Monocyte-derived macrophages	CS-induced PF↑	*In vivo*	(84)
SARS-CoV-2-	–	–	CD163+ monocyte-derived macrophages↑	SARS-CoV-2-induced PF↑	Patients	(85)
Viral and bacterial	Deficiency of TRIM29	Unknown	AMs↓	Type I interferons↑, less susceptible to the influenza virus	*In vivo*	(87)
Macrophage depletion	I/R	–	Macrophages↓	Renal fibrosis↓	*In vivo*	(95, 96)
High-fat diet feeding and treated with low-dose lipopolysaccharide	(Atg5^fl/fl^LysM-Cre+) mice	Macrophage autophagy↓	proinflammatory M1↑, anti-inflammatory M2↓	Inflammation↑	*In vivo*	(21)
USP19	–	Macrophage autophagy↑	Anti-inflammatory M2↑	Inflammation↓	*In vivo*	(24)
–	UUO mouse model	Macrophage mitophagy↑	Macrophage M1 polarization↓	Inflammation, renal fibrosis↓	*In vivo*	(100)
Rapamycin	–	Macrophage autophagy↑	Macrophage M1 polarization↓	Renal fibrosis↓	*In vivo* and *in vitro*	(102)
Quercetin	Obstructive mouse model	–	Macrophage M1 polarization↓	Renal fibrosis↓	*In vivo*	(103)
Repeated intraperitoneal injection of carbon tetrachloride	Specifically knock out the Atg5 in the myeloid lineage	Macrophage autophagy↓	–	Liver injury, chronic liver inflammation, and liver fibrosis↑	*In vivo*	(108)
Pharmacological and gene-level interventions to inhibit LAP	–	–	Controlling polarization of macrophages	liver inflammation, and liver fibrosis↑	*In vivo*	(113)
–	Myocardial infarction	–	CCR2+ macrophages	Cardiac fibrosis and heart failure	*In vivo*	(127)
Inhibition of TLR2	–	–	Macrophages↓	Ang II-induced cardiac fibrosis↓, inflammatory response↓	*In vivo*	(135)
Adiponectin	–	Macrophage autophagy↑	–	Ang II-induced cardiac fibrosis↓, inflammatory response↓	*In vivo* and *in vitro*	(136)

AM: alveolar macrophage; CS: crystalline silica; PF: pulmonary fibrosis; UUO: unilateral ureteral obstruction; I/R: ischemia/reperfusion; USP19: ubiquitin-specific protease 19; LAP: LC3-associated phagocytosis; CCR2-: C-C chemokine receptor type 2 negative; CCR2+: C-C chemokine receptor type 2 positive. ↑ means this biological process is activated. ↓ means this biological process is suppressed.

### Macrophage autophagy in lung fibrosis

As a common pathological feature and final outcome of many pulmonary diseases, pulmonary fibrosis (PF) is mainly characterized by excessive ECM accumulation in the lungs, which causes the thickening of the alveolar walls, and ultimately results in the destruction of alveolar structures and respiratory failure (70, 71). A common form of pulmonary fibrosis is idiopathic pulmonary fibrosis (IPF), which is characterized by progressive lung scarring and the histological picture of usual interstitial pneumonia, with increasing cough and dyspnoea (72). As a disease of aging, IPF affects about 3 million people worldwide, with the incidence increasing significantly with age (73). The dysfunction of type II alveolar epithelial cells is thought to be the starting factor of PF, which will then results in ECM overproduction *via* the activation of fibroblasts. Besides the type II alveolar epithelial cells, other cells, such as macrophages, also participate in the fibrotic process and play an essential role during this process (70, 74). According to their localization in the lungs, macrophages are classified into two types, alveolar macrophages (AMs) and interstitial macrophages (IMs) (75). Under normal conditions, AMs are located in the airspace of the alveoli and are the main cellular content of the alveoli.Therefore, they are known as the natural guardians of the respiratory tract and the fine control of their activation is essential to prevent inflammation and PF (76).

AMs play an essential role in silicosis, which is caused by exposure to crystalline silica (CS) particles and is characterized by chronic inflammation and PF (77). As the natural guardians of the respiratory tract, AMs can engulf the silica dust in the alveoli of the human body to prevent it from causing damage to other cells. However, CS swallowed by AM cannot be cleared by lysosomal digestion, causing the apoptosis of AMs in silicosis patients. The apoptosis of AMs will re-release the phagocytosed CS into the alveolar, triggering a new round of phagocytosis and apoptosis reaction, forming a vicious cycle, and eventually leading to persistent inflammation and PF (78). Considering the essential role of autophagy in inhibiting apoptosis and inflammation of AMs, it may play a protectiverole in the silicosis progression. Du et al. found that exposure to CS can trigger autophagy activity of AMs, which can protect AMs from CS-induced apoptosis (79). Trehalose, an activator of TFEB and the autophagy-lysosome biogenesis response, can alleviate apoptosis of AMs by protecting the autophagy-lysosomal function during the progression of silicosis (80, 81). As a steroidal saponin possessing many biological activities and health benefits, Dioscin was reported to have a protective effect against CS-induced PF in Mice (82). Further study showed that it can alleviate CS-induced Inflammation and PF by promoting autophagy of AMs (79). Mechanistically, Dioscin triggers the activity of AMs autophagy, which can reduce mitochondrial reactive oxygen species (mtROS) mass caused by CS, down-regulate the activation of mitochondria-dependent apoptosis pathway, and promote AMs survival, causing the reduced secretion of inflammatory factors and chemokines, and finally alleviating inflammation and PF (79). Notably, the protective effects of the dioscin disappeared in Atg5^flox/flox^Dppa3-Cre/+ mice, which specifically lack autophagy function in AMs *via* deleting Atg5 gene through Cre/loxP system (79). Additionally, microRNA-205-5p (miR-205-5p) has also been reported to inhibit CS-induced PF by promoting the AMs autophagy (83). These results suggest that tissue-resident macrophage (AMs) autophagy can inhibit cell apoptosis, inflammation, and PF during the silicosis progression.

In addition to the tissue-resident macrophages, the lung also has some monocyte-derived macrophages. Jessop et al. found that CS exposure could enhance the autophagic activity of mouse monocyte-derived macrophages (84). Specifically deleting Atg5 gene using LysM-Cre in mice (Atg5^fl/fl^LysM-Cre+) causes the impairment of autophagy in monocyte-derived macrophages, these transgenic mice were more sensitive to CS compared with littermate controls, shown as the elevated of inflammatory factors, such as IL-18, and the increased alarmin HMGB1 in the whole lavage fluid (84). Besides, these transgenic mice were more susceptible to spontaneous inflammation and disease, and more severe inflammation and PF when subjected to CS (84). These results suggest that monocyte-derived macrophage autophagy also plays a protective role in CS-induced inflammation and PF.

In addition to CS, some viruses, such as SARS-CoV-2, can also cause PF, and macrophages also play an important role in this process (85, 86). Wendisch et al. showed that SARS-CoV-2 infection can induce immunological and pathological changes in the lung of a patient, these changes are the typical characters of PF, and a subset of CD163+ monocyte-derived macrophages are responsible for this fibroproliferative acute respiratory distress (85). Xing et al. found that the E3 ubiquitin ligase TRIM29 was specifically expressed in AMs and can regulate AMs activation during the infection of viral and bacterial in the respiratory tract. Deficiency of TRIM29 can promote AMs to produce more type I interferons and make the mice lacking TRIM29 less susceptible to the influenza virus (87). Whether TRIM29 control AMs polarization and activation through the regulation of autophagy is still unknown. However, a recent study found that TRIM29 can promote autophagy in lung squamous cell carcinoma by activating BECN1 at the transcription level (88). Therefore, lack of TRIM29 may down-regulate the autophagy of AMs, which may promote AMs M1 polarization and activation to produce more inflammatory factors and type I interferons. Of course, more evidence is needed to prove this hypothesis.

**Figure 2:**
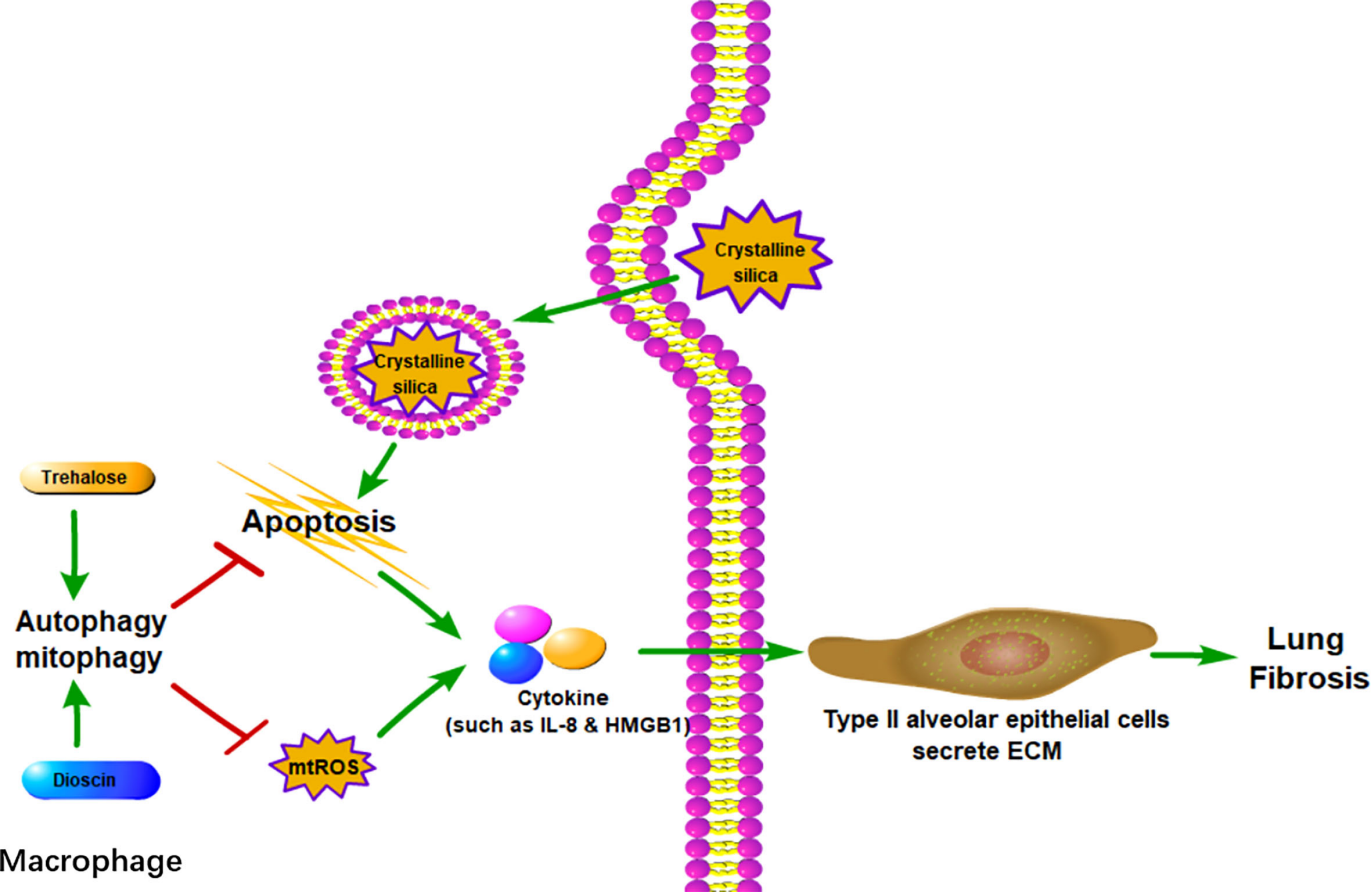
Macrophage autophagy alleviates lung fibrosis. In AMs, autophagy can reduce crystalline silica-associated apoptosis and mtROS, both of which could promote cytokines such as IL-8 and HMGB1. With the stimulation of cytokines, type II alveolar epithelial cells could secrete ECM to cause lung fibrosis. mtROS: mitochondrial reactive oxygen species.

In summary, macrophages, both AMs, and monocyte-derived macrophages play essential roles in lung inflammation and PF caused by various reasons. Various injury factors can cause M1 macrophage polarization and even cell death(**Figure 2**). As an important mechanism to maintain cell homeostasis, autophagy can inhibit M1 macrophage polarization and cell death by phagocytosis and digestion of invading pathogens or substances. From this perspective, macrophage autophagy can inhibit chronic inflammation and thereby inhibit PF. However, it should be noted that there are different views, which suggest that autophagy may aggravate lung injury and PF under certain circumstances, such as when it is too high or uncontrolled (77, 89).

### Macrophage autophagy in renal fibrosis

As the common final pathway of nearly all chronic and progressive kidney diseases to progress to end-stage renal failure, renal fibrosis refers to the accumulation of ECM in the renal parenchyma, which affects about 10% of the global population (90–92). The main function of the mammalian kidney is to keep the water, electrolyte, and acid-base balance of the body, while meanwhile excreting metabolic waste from the body. Fibrosis gradually leads to loss of these essential functions of the kidney, and eventually, the patients can only be kept alive by renal replacement therapy. After severe injury or multiple injuries, kidney tissue appears to have maladaptive repair, leading to chronic inflammation, which further promotes renal fibrosis. Macrophages play an essential role in chronic inflammation and the resulting renal fibrosis (93, 94). Ko et al. showed that severe ischaemic/reperfusion injury can lead to persistent inflammation and consequently the progression of renal fibrosis, whereas, macrophage depletion using liposome clodronate can alleviate inflammation and renal fibrosis caused by ischemia/reperfusion in a mouse model (95, 96). These results suggest that macrophages are indeed an important driver of persistent inflammation and renal fibrosis after ischemia/reperfusion, therefore, targeting macrophage infiltration or activation may be an effective way to prevent the development of chronic kidney disease after severe injury.

Autophagy can control the harmful effects of an infection and the consequent immune reaction through sequestration of pathogens, such as viruses, into autophagosomes and then delivery to the lysosomes for degradation (28). Recent studies have shown that autophagy can downregulate the proinflammatory response of macrophages (21). To study the effects of macrophage autophagy on inflammation, Liu et al. generated conditional knockout mice deleting Atg5 in macrophages using LysM-Cre, and found that transgenic mice showed systemic and hepatic inflammation after high-fat diet feeding and treated with low-dose lipopolysaccharide (21). Further study showed that loss of autophagy in macrophages led to abnormalities in macrophage polarization with the increasing of proinflammatory M1 and decreasing of anti-inflammatory M2, which caused an increase of inflammation (21). These findings suggest that macrophage autophagy plays an essential role in macrophage polarization and the downregulating of inflammation, therefore, targeting macrophage autophagy may be a potential way to inhibit chronic inflammation and the resulting renal fibrosis.

As an endoplasmic reticulum (ER)-anchored deubiquitinating enzyme, ubiquitin-specific protease 19 (USP19) is known to play an essential role in regulating ER-associated protein degradation, DNA damage repair, and in maintaining genome stability (97, 98). Recently, Liu et al. showed that USP19 can also restrain inflammation and promote macrophage polarization by regulating NLRP3 function through autophagy (24). Mechanistically, USP19 can increase autophagy flux and reduce the generation of mitochondrial reactive oxygen species, resulting in the inhibition of inflammatory responses and promotion of M2-like macrophage polarization (24). As the main place mammalian cells produce energy, mitochondria are constantly exposed to the high concentration of reactive oxygen species, causing them more vulnerable to mitochondrial DNA mutations and protein misfolding (99). To maintain a healthy and functional mitochondrial network, mammalian cells have evolved multiple quality control systems, mitophagy—cleaning dust particles and the injured mitochondria *via* autophagy—being one of them (99). Bhatia et al. recently reported that macrophage mitophagy can protect mouse kidney from fibrosis by regulating the PINK1/MFN2/Parkin-mediated pathway in two experimental kidney fibrosis mouse models (100). These results demonstrate that gene-level intervention to promote macrophage autophagy or macrophage mitophagy can inhibit macrophage M1 polarization and the resulting inflammation and renal fibrosis.

Rapamycin, an activator of autophagy by inhibiting mechanistic Target Of Rapamycin Complex 1 (mTORC1), can delay aging and extend lifespan in multiple organisms, and has been approved as an immuno-suppressant in 1999 by Food And Drug Administration (101). Zhang et al. found that lymphangiogenesis played an essential role in renal fibrosis, and the activation of macrophage autophagy by rapamycin can inhibit M1 macrophage polarization and the transdifferentiation of M1 macrophages into lymphatic endothelial cells, and the resulting lymphangiogenesis and renal fibrosis (102). Quercetin, a natural flavonoid compound, exists in the plant flowers, leaves, and fruit in the form of glycosides, and has been proven to have antioxidant and anti-inflammatory properties (103). Lu et al. found that administration of quercetin can mitigate mouse kidney injury and fibrosis by inhibiting M1 macrophage polarization in the obstructive mouse model (60). These results suggest that drug-level intervention of macrophage autophagy can affect its polarization and subsequently chronic inflammation and renal fibrosis.

**Figure 3:**
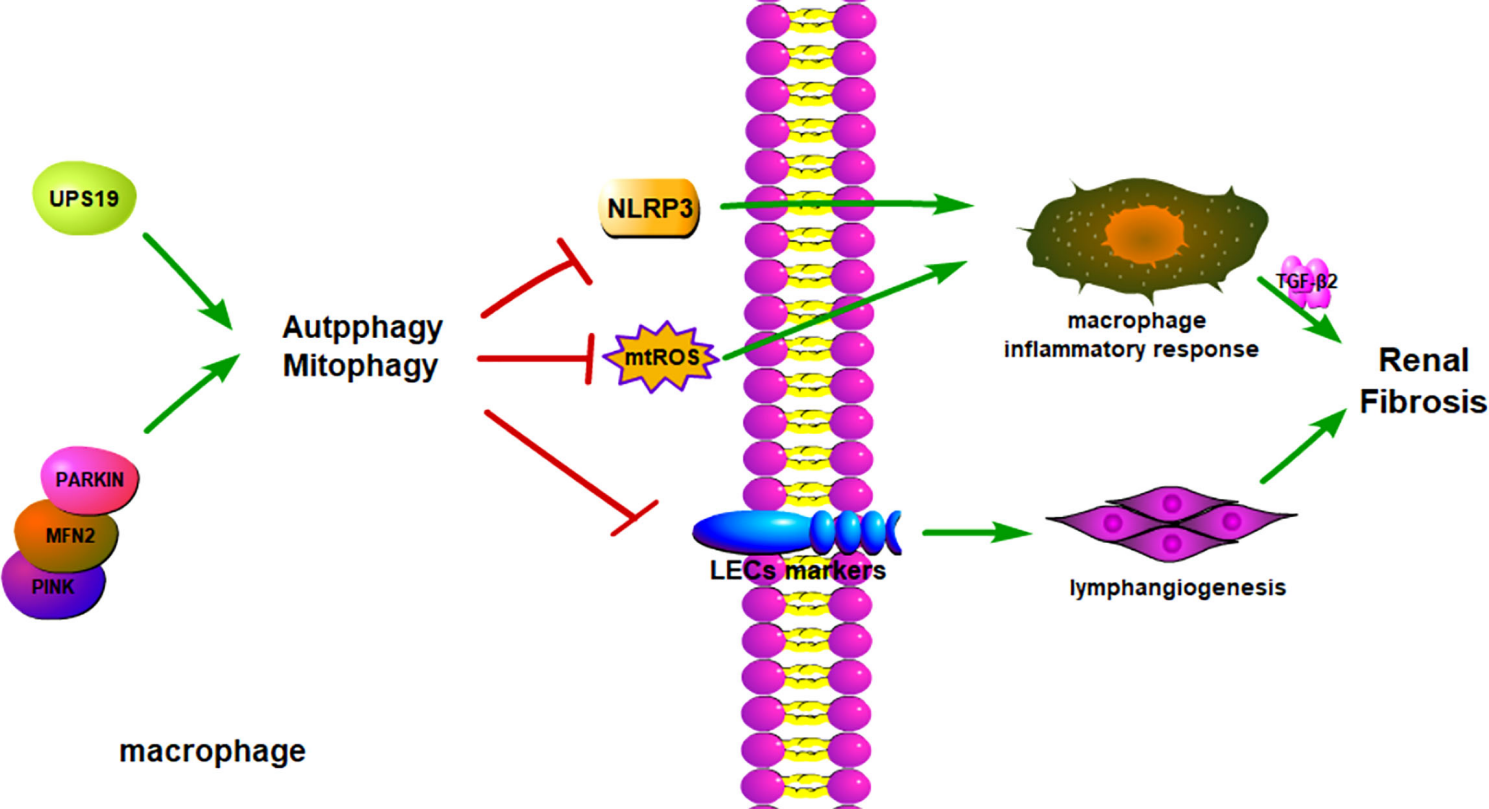
Macrophage autophagy alleviates kidney fibrosis. Besides secreting inflammatory cytokines, macrophages could transform into LECs to promote an inflammatory environment. Macrophage autophagy could reduce macrophage transformation into LECs and cytokines secretion. The autophagy regulation target is USP19 and PINK/MFN2 pathways, and it can influence the downstream proteins to change autophagy conditions. LECs, Lymphatic Endothelial Cell.

In summary, when the kidney is slightly injured, macrophages will infiltrate the damaged site and remove damaged or necrotic cells, helping the tissue to restore its original structure and function. However, when the kidney is severely or repeatedly injured, macrophages will massively infiltrate into the damaged site and persist, leading to chronic inflammation and renal fibrosis. Autophagy of macrophages can inhibit macrophage polarization to M1, thereby inhibiting inflammation and renal fibrosis (**Figure 3**). Therefore, targeting macrophage autophagy through gene or drug intervention is expected to be a potential therapeutic means to inhibit chronic inflammation and renal fibrosis. But at the same time, we should also recognize that macrophages are a very heterogeneous class of cells, there are many types of cells, and different types of cells will change each other (93). Different types of macrophages play different roles in disease progression, some even opposing roles. Enhancing macrophage autophagy can inhibit the polarization of macrophages to pro-inflammatory M1, but it may also promote the transformation of macrophages to profibrotic M2. M2 macrophages may promote renal fibrosis by secreting TGF-*β*1. Therefore, when enhancing macrophage autophagy at the gene or drug level for the treatment of renal fibrosis, the possible side effects should be fully considered. On the other hand, how to accurately target and deliver drugs to macrophages are also big problems we are facing at present (104).

### Macrophage autophagy in liver fibrosis and cardiac fibrosis

As the common pathological outcome of various chronic liver diseases, liver fibrosis refers to the excessive accumulation of extracellular matrix proteins in the liver, leading to liver parenchyma gradually being replaced by scar tissue, and liver function gradually being lost (105). The end stage of liver fibrosis is cirrhosis, which is a major cause of morbidity and mortality worldwide due to the severe complications of portal hypertension and liver failure and the high risk of an incident of hepatocellular carcinoma (106). Chronic liver injuries, such as hepatotoxic injury and cholestatic injury, result in chronic liver inflammation and the resulting liver fibrosis (105). Increasing evidence shows that macrophage activation and polarization play an essential role in liver inflammation and liver fibrosis (107). Therefore, controlling systemic and liver inflammation by targeting monocytes/macrophages is a potential strategy to inhibit liver fibrosis and its progression to cirrhosis.

Considering the essential role of macrophage autophagy in regulating innate immunity and resultant tissue inflammation, the intervention of macrophage autophagy may be a good means to inhibit liver inflammation and liver fibrosis. Ilyas et al. showed that macrophage autophagy can down-regulate hepatic inflammation by inhibiting the production of inflammasome-dependent IL-1*β* (108). By using the Cre-loxP system to specifically knock out the Atg5 in the myeloid lineage, Lodder et al. explored the function of macrophage autophagy in chronic liver injury in a mouse model by repeated intraperitoneal injection of carbon tetrachloride, they found that macrophage autophagy played a protective role in liver injury, chronic liver inflammation, and liver fibrosis by inhibiting the secretion of IL1A and IL1B. These results suggest that macrophage autophagy indeed can alleviate liver injury, liver inflammation and fibrosis in a drug-induced liver injury mouse model.

In addition to the classical autophagy pathway, LC3-associated phagocytosis (LAP), a novel form of non-canonical autophagy, also has been reported to play an essential role in regulating immune response and inflammation *via* controlling the polarization of macrophages (109). Macrophages can clear extracellular particles, such as apoptotic cells and pathogens, through LAP. In simple terms, macrophages can bind with dead cells *via* receptors present on their surface, which causes autophagy machinery to be translocated to the phagosome, and subsequently LC3 conjugation (110). Through the LAP of macrophages, the apoptotic cells or *β*-amyloid can be rapidly removed, thus reducing inflammation of damaged tissue (111, 112). Recently, Wan et al. found that LAP indeed can inhibit inflammation and liver fibrosis, as both pharmacological and gene-level interventions to inhibit LAP can aggravate inflammatory and fibrotic phenotypes (113). Therefore, targeting LAP to inhibit inflammation and fibrosis may be a promising therapeutic strategy to treat patients with chronic liver disease.

Myocardial infarction or pressure overload can lead to cardiac remodeling. During the myocardial infarction-induced remodeling process, cardiac fibrosis appears in the infarcted areas of the myocardium to maintain the structure of the heart (114–116). Besides, cardiac fibrosis also occurs in the pressure overload-induced cardiac remodeling process, resulting in the progression of heart failure with preserved ejection fraction (117). In both cases, the degree of interstitial fibrosis was associated with mortality and major adverse cardiovascular events in patients with heart failure (118–121). The severity of cardiac fibrosis and heart failure is closely related to the degree of inflammation (122–124), and macrophages, the important immune cells in innate immunity, play an essential role in the process of cardiac fibrosis (107).

Under normal physiological conditions, at least two macrophage subsets exist in the heart: C-C chemokine receptor type 2 negative (CCR2-) and CCR2 positive (CCR2+) macrophages (125). CCR2- macrophages are resident macrophages that are derived from embryonic progenitors (yolk sac and fetal liver), whereas, CCR2+ macrophages are monocyte-derived macrophages that are derived from adult bone marrow progenitors (126). CCR2- and CCR2+ macrophages have a distinct function during cardiac fibrosis, with the CCR2- macrophages facilitating tissue repair, while CCR2+ macrophages promote tissue inflammation. After myocardial infarction, Ly6C^high^, CCR2+ monocytes infiltrate into the heart and differentiate into CCR2+ macrophages to promote pro-inflammatory responses, collateral tissue damage, and ultimately lead to cardiac fibrosis and heart failure (127). Therefore, it is now generally accepted that monocyte-derived infiltrating macrophages can promote fibrosis *via* promoting cardiac inflammation, while cardiac resident macrophages can inhibit cardiac fibrosis by facilitating cardiac repair (128–130).

Autophagy of cardiomyocytes plays an essential role in cardiac homeostasis and function (131, 132). Autophagy of cardiomyocytes can maintain cardiac structure and function under baseline conditions and can alleviate cardiac injury under most stressed conditions (131, 133). Besides, autophagy can also inhibit chronic ischemic remodeling and promote cardiac adaptation to pressure overload by reducing misfolded protein, mitochondrial damage, and oxidative stress (131, 134). However, most of the studies regarding cardiac autophagy are focused on cardiomyocytes, whereas the nonmyocyte, such as macrophage, is poorly understood. Qi et al. showed that inhibition of TLR2 can inhibit Ang II-induced cardiac fibrosis by attenuating macrophage recruitment and the inflammatory response in the heart (135), whereas, Adiponectin can promote macrophage autophagy *via* the adenosine 5’-monophosphate-activated protein kinase pathway and inhibit Ang II-induced inflammatory responses and the resulting cardiac fibrosis (136).

**Figure 4:**
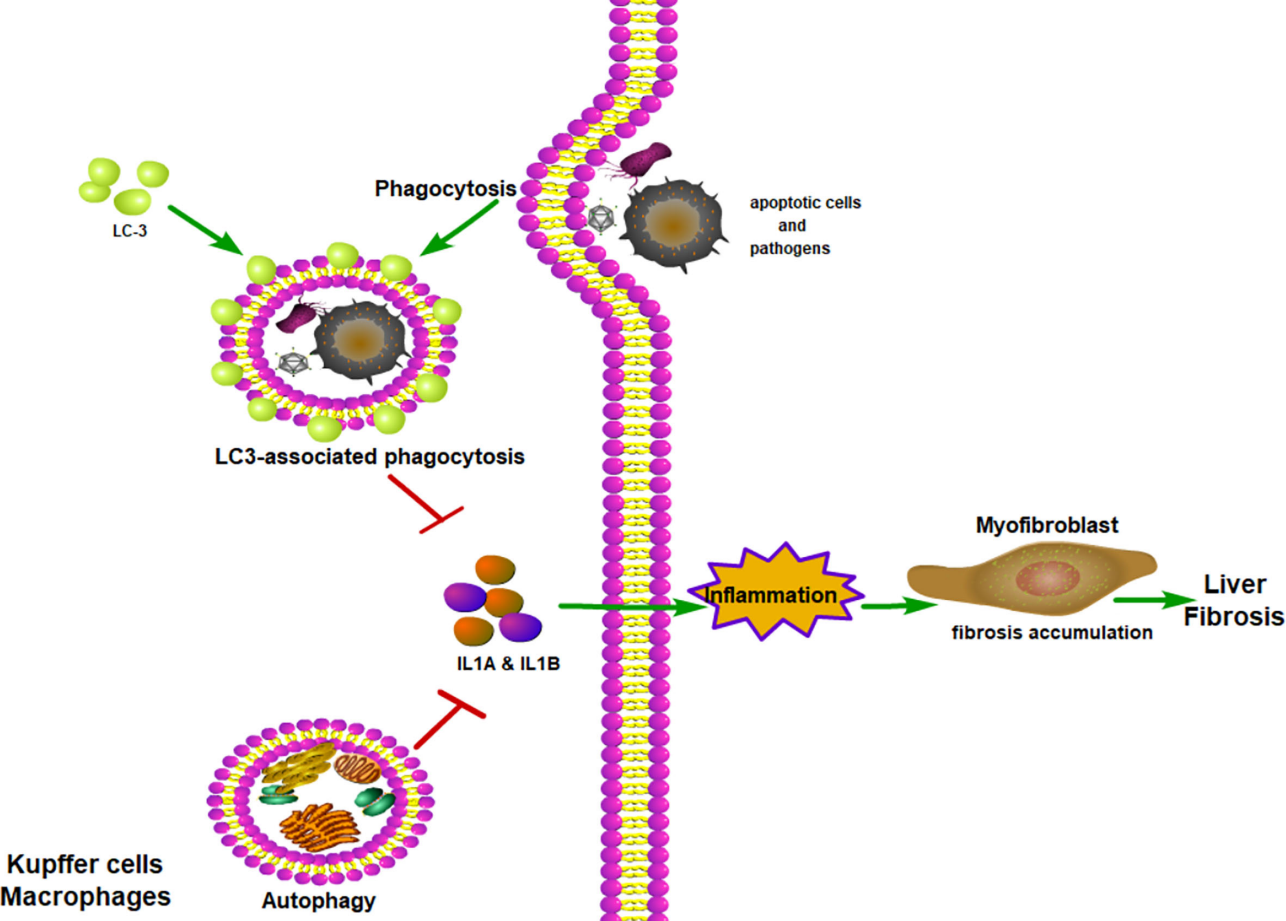
Macrophage autophagy alleviates liver fibrosis. LC-3-associated phagocytosis could remove apoptotic cells and pathogens to reduce inflammation. With the assistance of LC-3-associated phagocytosis, autophagy could reduce inflammatory cytokines being secreted. With fewer inflammatory cytokines, myofibroblast would produce less collagen and relieve lung fibrosis. ITAMi, inhibitory immunoreceptor tyrosine-based activation motif; LAP, LC3-associated phagocytosis.

**Figure 5:**
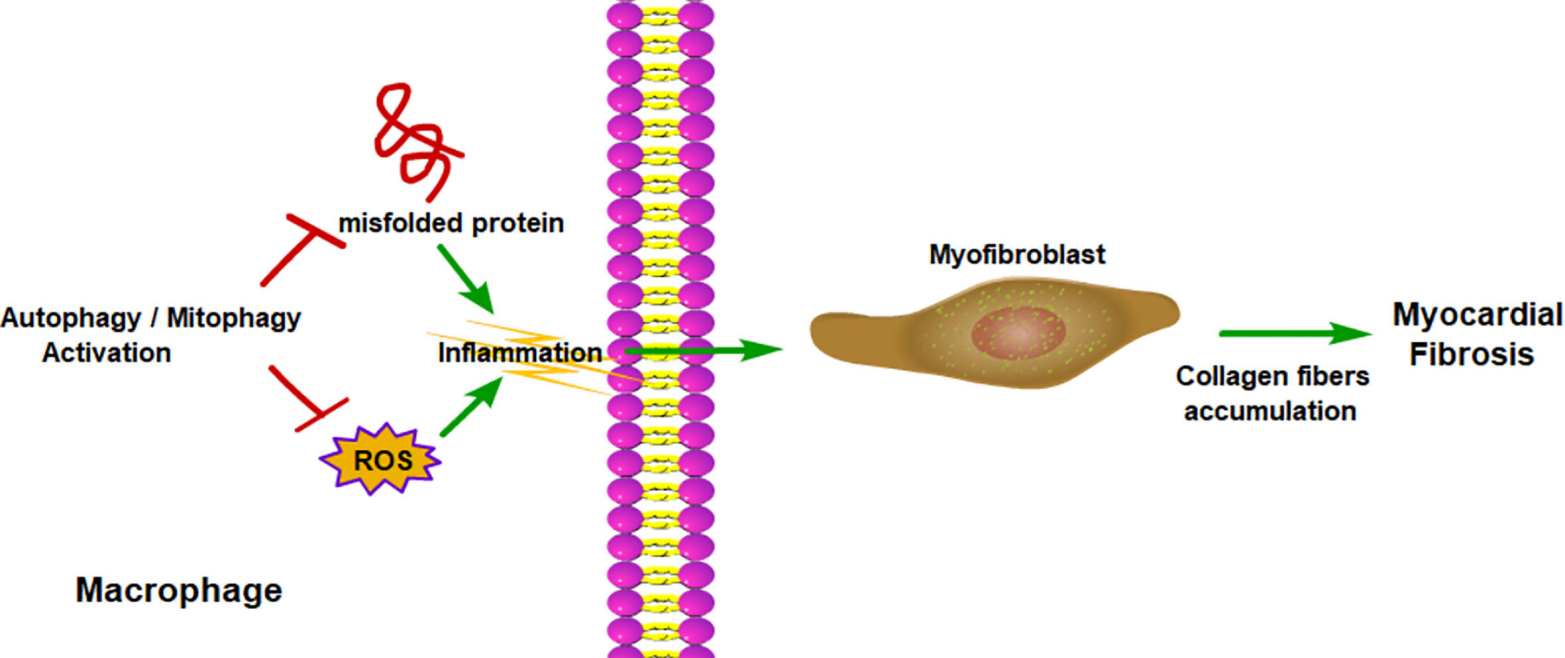
Macrophage autophagy alleviates myocardial fibrosis. Macrophage autophagy reduces the production of ROS and removes misfolded protein, which promote inflammation. With less inflammatory cytokines, myofibroblast would produce less collagen and relieve myocardial fibrosis. ROS, reactive oxygen species.

In summary, chronic liver injuries can induce inflammation, which can promote liver fibrosis. Macrophage autophagy, both classical autophagy and LAP, can inhibit inflammation, therefore mitigate liver fibrosis (**Figure 4**). Myocardial infarction or pressure overload can result in cardiac remodeling and cardiac fibrosis. Inflammation also plays an important role in cardiac fibrosis. The role of macrophage autophagy in cardiac fibrosis is rarely studied and its function is still unknown. Only a few studies have shown that monocyte-derived macrophage autophagy seems to reduce cardiac inflammation and fibrosis (136) (**Figure 5**). Therefore, more studies are needed to prove the role of macrophage autophagy, especially the resident macrophage autophagy in cardiac fibrosis.

## Conclusions and perspectives

As illustrated in this review, macrophage autophagy can protect organs from chronic inflammation and organ fibrosis. Nevertheless, it remains unclear how autophagy affects macrophage polarization. Besides, as mentioned above, there are many types of autophagy. Our current research on macrophage autophagy mainly focuses on macroautophagy, while the role of other types of autophagy, such as chaperone-mediated autophagy, microautophagy, and various selective autophagy in macrophage polarization, chronic inflammation, and organ fibrosis are still poorly understood. Thus, in future work, it will be important to study the mechanism of autophagy in the regulation of macrophage polarization, elucidating the function of other types of autophagy in macrophage polarization, chronic inflammation, and organ fibrosis.

Macrophages are highly dynamic and heterogeneous cells, there are many types of macrophages in tissues, and different types of macrophages may perform different functions (137). For example, infiltrating macrophages and tissue-resident macrophages may play different roles in organ fibrosis (138, 139), and it is still unknown whether autophagy has different roles in the polarization of different types of macrophages. Next, we should use more advanced technologies, such as single-cell RNA/protein sequencing (140–142), to further clarify the types of macrophages in different tissues under different pathological conditions and to further study the role of autophagy in their polarization, chronic inflammation and organ fibrosis.

Finally, it is important to note that a lot of the research we are doing now is done on mice, using the disease model of mice to simulate the disease state of humans. After all, we cannot directly use humans to do *in vivo* gene editing experiments. But the results of experiments with mice may not apply to humans. In the future, it may be necessary to use 3D culture to grow human organs (organoids) *in vitro* (143–147), and conduct experiments on these organoids, so that the conclusions may be more valuable for reference.

## Author contributions

JW, LD-Y, SL, TJ-X and LH-F designed and wrote this review. CY reviesd this review. All authors contributed to the article and approved the submitted version.

## Funding

This work was supported by grants from the National Natural Science Foundation of China (81974095) and the Natural Science Foundation of Guangdong Province (2019A1515110152).

## Conflict of interest

The authors declare that the research was conducted in the absence of any commercial or financial relationships that could be construed as a potential conflict of interest.

## Publisher’s note

All claims expressed in this article are solely those of the authors and do not necessarily represent those of their affiliated organizations, or those of the publisher, the editors and the reviewers. Any product that may be evaluated in this article, or claim that may be made by its manufacturer, is not guaranteed or endorsed by the publisher.
